# Noninvasive Ventilation During Immediate Postoperative Period in Cardiac Surgery Patients: Systematic Review and Meta-Analysis

**DOI:** 10.21470/1678-9741-2017-0032

**Published:** 2017

**Authors:** Suzimara Monteiro Pieczkoski, Ane Glauce Freitas Margarites, Graciele Sbruzzi

**Affiliations:** 1 Multi-Professional Integrated Residency in Health and Cardiovascular Care of the Hospital de Clínicas of Porto Alegre (HCPA), Porto Alegre, RS, Brazil.; 2 Hospital de Clínicas of Porto Alegre (HCPA), Porto Alegre, RS, Brazil.; 3 Universidade Federal do Rio Grande do Sul (UFGRS), Porto Alegre, RS, Brazil.

**Keywords:** Thoracic Surgery, Cardiovascular Surgical Procedures, Noninvasive Ventilation, Meta-Analysis

## Abstract

**Objective:**

To verify the effectiveness of noninvasive ventilation compared to conventional physiotherapy or oxygen therapy in the mortality rate and prevention of pulmonary complications in patients during the immediate postoperative period of cardiac surgery.

**Methods:**

Systematic review and meta-analysis recorded in the International Prospective Register of Ongoing Systematic Reviews (number CRD42016036441). The research included the following databases: MEDLINE, Cochrane Central, PEDro, LILACS and manual search of the references of studies published until March 2016. The review included randomized controlled trials with patients during the immediate postoperative period of cardiac surgery, which compared the use of noninvasive ventilation, BiLevel modes, continuous positive airway pressure, intermittent positive pressure breathing and positive pressure ventilation with conventional physiotherapy or oxygen therapy, and assessed the mortality rate, occurrence of pulmonary complications (atelectasis, pneumonia, acute respiratory failure, hypoxemia), reintubation rate, ventilation time, time spent in the intensive care unit (ICU), length of hospital stay and partial pressure of oxygen.

**Results:**

Among the 479 selected articles, ten were included in the systematic review (n=1050 patients) and six in the meta-analysis. The use of noninvasive ventilation did not significantly reduce the risk for atelectasis (RR: 0.60; CI95% 0.28-1.28); pneumonia (RR: 0.20; CI95% 0.04-1.16), reintubation rate (RR: 0.51; CI95%: 0.15-1.66), and time spent in the ICU (-0.04 days; CI95%: -0.13; 0.05).

**Conclusion:**

Prophylactic noninvasive ventilation did not significantly reduce the occurrence of pulmonary complications such as atelectasis, pneumonia, reintubation rate and time spent in the ICU. The use is still unproven and new randomized controlled trials should be carried out.

**Table:** 

Abbreviations, acronyms & symbols				
ARF	= Acute respiratory failure		IS	= Incentive spirometer
CABG	= Coronary artery bypass grafting		NIV	= Noninvasive ventilation
CP	= Conventional physiotherapy		PaO_2_	= Partial pressure of oxygen
CPAP	= Continuous positive airway pressure		PO	= Postoperative
CPB	= Cardiopulmonary bypass		PSV	= Positive pressure ventilation
ICU	= Intensive care unit		RCTs	= Randomized controlled trials
IPPB	= Intermittent positive pressure breathing			

## INTRODUCTION

Patients in the postoperative (PO) period of cardiac surgery have greater risk to develop pulmonary complications. These complications can increase hospitalization time, morbidity, mortality, and costs for the health system^[1]^. Among the most frequent pulmonary complications are atelectasis, pneumonia, pulmonary edema and acute respiratory failure (ARF). Atelectasis is one of the most common^[1,2]^.

The etiology of pulmonary complications results from a multifactorial process. Surgical factors such as the use of cardiopulmonary bypass (CPB), anesthesia, surgery time, mechanical ventilation time, pleural opening, phrenic nerve alteration, use of the mammary artery in myocardial revascularization surgery, pain in the sternal surgical wound and in the surgical drains lead to a decrease in the functional residual capacity and increase of intrapulmonary shunt^[3-6]^. In addition, preoperative factors regarding the patient, such as previously existing lung diseases, smoking, old age, poor nutritional health, among others, are a predisposition to complications^[7]^.

Certain measures are used during the PO of cardiac surgeries, in an attempt to minimize pulmonary complications, such as adequate analgesia, oxygen therapy and physiotherapy. The physiotherapist uses the resources and chest physiotherapy techniques, such as deep breathing stimulation, cough stimulation, use of incentive spirometers, early patient mobilization and ambulation^[1,8]^. However, sometimes these features and techniques are not enough, and additional measures, such as the use of noninvasive ventilation (NIV), are necessary.

NIV is a support for spontaneous ventilation with portable ventilators. Its use as a prophylactic measure aims to reduce the incidence of endotracheal intubation, length of hospital stay and prevent pulmonary complications^[9,10]^. However, even with randomized controlled trials (RCTs) and a systematic review, there is no consensus in the literature regarding its use as a prophylactic measure after cardiac surgery.

Zarbock et al.^[10]^ carried out a study with 468 elective heart surgery patients during the period of postoperative care and showed that the use of prophylactic continuous positive airway pressure (CPAP) reduced the incidence of pulmonary complications such as hypoxemia, pneumonia, reintubation rate, and reduced the readmission rate in intensive care, compared to the control group. However, another study with 30 patients undergoing coronary artery bypass grafting (CABG) showed that CPAP therapy minimized the decrease in partial pressure of oxygen (PaO_2_) after extubation, however, it was unable to prevent the decrease of oxygenation on the second PO day^[11]^. In 2011, a systematic review investigated the use of NIV as a preventive measure in patients undergoing heart surgery, including four studies. The authors found that NIV, compared to standard treatment with oxygen therapy and chest physiotherapy, significantly improved gas exchange without any significant difference in the rate of atelectasis^[1]^.

Thus, the existence of new RCTs related to the prophylactic use of NIV in patients during the immediate PO period of cardiac surgery, and the absence of meta-analysis, justify a systematic review with a recent meta-analysis on the subject. Thus, the objective of this review is to verify the effectiveness of the use of NIV compared to conventional physiotherapy (CP) or oxygen therapy in the mortality rate and prevention of pulmonary complications in patients in the immediate PO of cardiac surgery.

## METHODS

This is a systematic review and meta-analysis of RCTs, registered with the International Prospective Register of Ongoing Systematic Reviews (PROSPERO) under the number CRD42016036441, and following the recommendations of the Preferred Reporting Items for Systematic Reviews and Meta-Analyses: The PRISMA Statement^[12]^ and the Cochrane Collaboration^[13]^.

### Eligibility Criteria

The review included RCTs with patients during the immediate PO period of heart surgery (CABG, valve replacement, among others) that compared the use of NIV, BiLevel, CPAP, intermittent positive pressure breathing (IPPB) and positive pressure ventilation (PSV) with CP or oxygen therapy. And also, that assessed mortality rate and incidence of pulmonary complications (atelectasis, pneumonia, ARF, hypoxemia) as primary outcomes, and reintubation rate, ventilation time, time spent in the intensive care unit (ICU), length of hospital stay and PaO_2_ as secondary outcomes. Studies that included patients in the PO period of other types of surgery, and patients who were heart transplant recipients, were excluded from the review.

### Search Strategy

The studies were found using a systematic search in the databases MEDLINE (via PubMed), Cochrane Central, PEDro, LILACS, in addition to a manual search of the references of published studies on the subject. There was no restriction of date and language for this research. The search included studies published from the start of the databases until March, 2016, and comprised the key descriptors and synonyms terms referring to "cardiac surgery", "coronary artery bypass", "tricuspid valve replacement", "mitral valve replacement", "aortic valve replacement", "noninvasive ventilation", "continuous positive airway pressure", and "positive-pressure respiration", combined with a sensitive list of ECR search terms developed by Robinson & Dickersin^[14]^. The complete search strategy used for PubMed is shown on Table 1. Other strategies will be available upon request.

**Table 1:** Research strategy used on PubMed.

#1	"Cardiac Surgery"[MeSH] OR "Cardiac Surgery" OR "Surgery, Thoracic" OR "Surgery, Cardiac" OR "Surgery, Heart" OR "Heart Surgery" OR "Procedure, Cardiac Surgical" OR "Procedures, Cardiac Surgical" OR "Surgical Procedure, Cardiac" OR "Surgical Procedures, Cardiac" OR "Surgical Procedures, Heart" OR "Cardiac Surgical Procedure" OR "Heart Surgical Procedures" OR "Procedure, Heart Surgical" OR "Procedures, Heart Surgical" OR "Surgical Procedure, Heart" OR "Heart Surgical Procedure"
#2	"Coronary Artery Bypass"[Mesh] OR "Coronary Artery Bypass" OR "Artery Bypass, Coronary" OR "Artery Bypasses, Coronary" OR "Bypasses, Coronary Artery" OR "Coronary Artery Bypasses" OR "Coronary Artery Bypass Surgery" OR "Bypass, Coronary Artery" OR "Aortocoronary Bypass" OR "Aortocoronary Bypasses" OR "Bypass, Aortocoronary" OR "Bypasses, Aortocoronary" OR "Bypass Surgery, Coronary Artery" OR "Coronary Artery Bypass Grafting"
#3	"Tricuspid Valve Replacement" OR "Tricuspid Valve Surgery" OR "Valve Replacement" OR "Valve Surgery" OR "Mitral Valve Replacement" OR "Mitral Valve Surgery" OR "Aortic Valve Replacement" OR "Aortic Valve Surgery"
#4	#1 OR #2 OR #3
#5	"Noninvasive ventilation"[MeSH] OR "Noninvasive Ventilation" OR "Noninvasive Ventilations" OR "Ventilation, Noninvasive" OR "Ventilations, Noninvasive" OR "Non-Invasive Ventilation" OR "Non-Invasive Ventilations" OR "Ventilation, Non-Invasive" OR "Ventilations, Non-Invasive" OR "Non Invasive Ventilation" OR "Non Invasive Ventilations" OR "Ventilation, Non Invasive" OR "Ventilations, Non Invasive" OR "bilevel ventilation" OR "Noninvasive Positive Pressure Ventilation" OR "CPAP" OR "BIPAP"
#6	"Continuous Positive Airway Pressure"[Mesh] OR "Continuous Positive Airway Pressure" OR "CPAP Ventilation" OR "Ventilation, CPAP" OR "Biphasic Continuous Positive Airway Pressure" OR "Bilevel Continuous Positive Airway Pressure" OR "Nasal Continuous Positive Airway Pressure" OR "Ncpap Ventilation" OR "Ventilation, ncpap"
#7	"Positive-Pressure Respiration"[Mesh] OR "Positive-Pressure Respiration
#8	#5 OR #6 OR #7
#9	(randomized controlled trial[pt] OR controlled clinical trial[pt] OR randomized controlled trials[mh] OR random allocation[mh] OR double-blind method[mh] OR single-blind method[mh] OR clinical trial[pt] OR clinical trials[mh] OR ("clinical trial"[tw]) OR ((single*[tw] OR double*[tw] OR OR triple*[tw]) AND (mask*[tw] OR blind*[tw])) OR ("latin square"[tw]) OR placebos[mh] OR placebo*[tw] OR random*[tw] OR research design[mh:noexp] OR follow-up studies[mh] OR prospective studies[mh] OR cross-over studies[mh] OR control*[tw] OR prospective*[tw] OR volunteer*[tw]) NOT (animal[mh] NOT human[mh])
#10	#4 AND #8 AND #9

### Study Selection

Two assessors (S.M.P and A.F.M) independently analyzed the titles and abstracts of the articles identified by the search strategy, strictly adhering to the inclusion and exclusion criteria. Articles that did not provide enough information in the titles and abstracts, were fully read by the same assessors, independently. The selection was made following the eligibility criteria. Disagreements over the inclusion of the studies were resolved by consensus among the assessors.

### Data Extraction

The data extraction was performed independently by the same two assessors, using a standardized form. Information on patient characteristics, intervention, outcomes, and methodological quality were extracted. Disagreements were resolved by consensus. The main outcomes were mortality rate and incidence of pulmonary complications (atelectasis, pneumonia, ARF, hypoxemia) and the secondary outcomes were reintubation rate, ventilation time (hours), time spent in the ICU, length of hospital stay (days), and PaO_2_.

### Risk of Bias Assessment

The methodological quality was evaluated independently by the same two assessors, in a descriptive manner, based on the recommendations of the Cochrane Collaboration^[13]^. The following items were evaluated: random sequence generation, allocation concealment, patient blinding, blinding of therapists and outcome assessors, intention-to-treat analysis, and description of losses and exclusions. These characteristics were considered as "not informed" in studies without clear description of them.

### Data Analysis

The data analysis was performed in a descriptive and quantitative manner. Regarding the categorical outcomes, relative risk and 95% confidence intervals were calculated using the random effect model (Mantel-Haenszel) according to the number of events reported in the intention-to-treat analysis of the original studies. For the continuous outcomes, the effect estimations were obtained by the difference between the averages and their standard deviations, with 95% confidence intervals using the random effect model. The statistical heterogeneity of the treatment effects between studies was assessed using the I-square inconsistency test, where values above 25% and 50% were considered as indicative of moderate and high heterogeneity, respectively. All analyses were carried out using the Review Manager software, version 5.3 (Cochrane Collaboration).

## RESULTS

### Description of the Studies

**Fig. 1:**
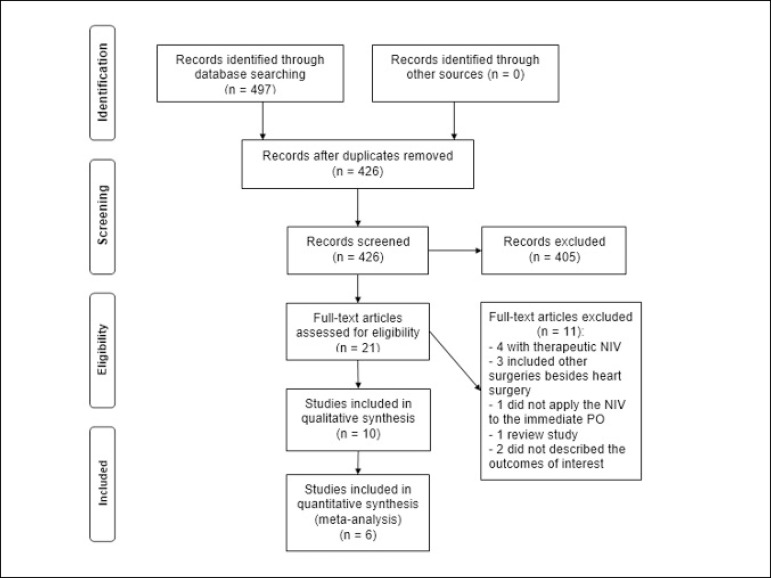
Flowchart of the studies included in the systematic review and meta-analysis. NIV=noninvasive ventilation. PO=postoperative

Four hundred and seventy-nine articles were identified with the search strategy, of which 21 studies were considered for detailed analysis. After the analysis, ten articles met the eligibility criteria and were included in this review, with a total of 1050 patients. Among these studies, three used the BiLevel mode^[8,15,16]^, four the CPAP^[10,11,17,18]^, one the BiLevel mode and CPAP in the same study^[3]^, one the IPPB^[19]^ and one the PSV^[20]^. Regarding the control groups, seven studies^[3,10,15-19]^ performed CP. Of these, one performed only CP^[16]^; the other used CP associated with incentive spirometer (IS)^[3,19]^, standard treatment (oxygen therapy and CPAP for some patients, pharmacological treatment)^[10]^, usual care (pharmacological measures and IS)^[15]^ and oxygen therapy^[17,18]^. Two studies^[8,11]^ received oxygen therapy exclusively and one of the studies^[20]^ did not clearly describe the comparison. Figure 1 shows the flowchart of the included studies and Table 2 the characteristics of these studies.

**Table 2:** Characteristics of the studies included in the review.

Study, year	Type of surgery	Participants (N - I/C)	Intervention vs. control groups	Intervention group	Control group	Assessed outcomes	Results
Al Jaaly et al.^[15]^, 2013	CABG	126 - 63/63	BiLevel+ usual care^a^, *vs.* usual care^a^+ CPAP for six patients who needed ventilation support	Stratified by BMI: BMI<30: IPAP 12 cmH_2_O, EPAP 5 cmH_2_O; BMI≥30: IPAP 17, EPAP 10 cmH_2_O; for 24h (removed so the patient could eat, or before if they could not tolerate it)	2x/day on the first two to three days after surgery	Mortality rate Atelectasis Pneumonia Reintubation rate MV Time Time spent in the ICU Length of hospital stay until release	Mortality rate: the same in both groups Atelectasis, pneumonia and Reintubation rate: less frequent in the intervention group MV Time: similar between groups (without statistically significant data) Time spent in the ICU: shorter in the intervention group, but without significant difference ( *P*=0.306) between groups. Length of hospital stay until release: shorter in the intervention group, with significant difference ( *P*=0.019) between groups, if the medical release is considered; and similar between groups, without significant difference (*P*=0.552) between groups, if non-medical factors are considered
Franco et al.^[16]^, 2011	CABG	26 - 13/13	BiLevel+ CP^b^*vs.* CP	IPAP: 8 to 12 cmH_2_O; EPAP: 6 cmH_2_O, 2x/day for 30 minutes	2x/day, 2 days after surgery	Atelectasis Length of hospital stay until release	Atelectasis: lower in the intervention group, without significant difference (P=0.08) between groups Length of hospital stay: shorter in the intervention group (without statistically significant data)
Jousela et al.^[11]^, 1994	CABG	30 - 15/15	CPAP *vs.* oxygen therapy	CPAP 7.4 cmH_2_O FiO_2 _0.3 for 8h	FiO_2_0.3 for 8h	AtelectasisPaO_2_	Atelectasis: similar between groups PaO_2:_better in the intervention group_,_with significant difference between groups (*P*<0.05)
Lopes et al.^[8]^, 2008	CABG or valve surgery	100 - 50/50	BiLevel *vs.* oxygen therapy (nasal catheter)	For 30 minutes, IPAP for generating a VC > 5 ml/kg (average value 10±2.12 cmH_2_O), EPAP 5 cmH_2_O, and oxygen attached to the mask at 5 l/min or enough for SpO_2_> 95%	5 l/min	PaO_2_MV Time	PaO_2_: better in the intervention group, with significant difference (*P*=0.0009) between groups MV Time: similar between groups, without significant difference ( *P* =0.526) between groups
Matte et al.^[3]^, 2000	CABG	96 33,33/30	CPAP + CP (coughing, exercises, aerosol therapy, mobilization) or BiLevel + CP *vs.* CP + IS (volume)	CPAP 5 cmH_2_O (1h/3h); BiLevel IPAP 12 cmH_2_O, EPAP 5 cmH_2_O (1h/3h)	CP parameters not described;IS 20/2 h	AtelectasisPaO_2_Time spent in the ICU	Atelectasis: less in the intervention group (without statistically significant data) PaO_2:_increased in the intervention group, but without significant difference *P*<0.01Time spent in the ICU: similar, without significant difference between groups
Mazullo, et al.^[20]^, 2010	CABG, valve replacement,Combined surgeries, Interatrial communication, aneurysm repair	32 - 14/18	NIV (PSV) *vs.*not described	PSV PEEP 5 cmH_2_O; levels of PSV adjusted to reach a current volume of 5 to 8 ml/kg; FiO_2_40%, for 2h	Not described	ARF after extubation	ARF: control group presented higher incidence. (without statistically significant data)
Oikkonenet al.^[19]^, 1991	CABG	52 - 26/26	IPPB+CP (chest physiotherapy techniques) *vs.* IS (volume) + CP	Airway peak pressure 10 to 15 cmH_2_O at least four times/day, minimum of 10 satisfactory inspirations, five to 10 minutes each session	1x/day, more frequently, if necessary CP	AtelectasisPaO_2_	Atelectasis: less in the intervention group; without significant difference between groups (*P*>0.1) PaO_2:_similar values between groups on the first three days
Pinilla et al.^[17]^, 1990	CABG	58 - 32/26	CPAP+ CP (chest physiotherapy) *vs.* oxygen therapy + CP	Between 5 and 7.5 cmH_2_O, for 12h	Not described	Atelectasis Hypoxemia (PaO_2_/FiO_2_)Time spent in the ICU	Atelectasis: not different between groups Hypoxemia (PaO_2_/FiO_2_)_:_ significantly improved ratio in the intervention group, (*P*<0.05), half an hour until 24h after extubation; after that, a decrease could be noted in both groupsTime spent in the ICU: similar between groups, without significant difference between groups
Thomas et al.^[18]^, 1992	CABG	28 - 14/14	CPAP+CP *vs.* oxygen therapy + CP	5 cmH_2_O, for 1h	Not described	Hypoxemia	Hypoxemia: significantly reduced the pulmonary shunt in the intervention group (*P*=0.016)
Zarbock et al.^[10]^, 2009	CABG or heart valve replacement	468 - 232/236	CPAP *vs.* standard treatment^c^	10 cmH_2_O, for at least 6h	Intermittent CPAP for 10 min every 4h at 10 cm H_2_O; other information was not described	Hypoxemia (PaO_2_/FiO_2 _<100)Nosocomial PneumoniaReintubation rate MV TimeTime spent in the ICU and at the hospital	Hypoxemia (PaO_2_/FiO_2_<100), pneumonia, reintubation rate: lower in the intervention group, with significant difference between groups (*P*=0.03) MV Time late extubation group: similar between groups, without significant difference ( *P*>0.05)Time spent in the ICU and at the hospital:similar between groups; without significant difference(*P*>0.05) between groups

CPAP: continuous positive airway pressure, MRS: myocardial revascularization surgery, EPAP: expiratory positive airway pressure, CP: conventional physiotherapy, I/C: intervention/control, BMI: body mass index, IPAP: inspiratory positive airway pressure, IPPB: intermittent positive pressure breathing, ARF: acute respiratory failure, IS: incentive spirometer, LLs: lower limbs, ULs: upper limbs, PaO_2_/FiO_2_: partial pressure arterial oxygen/fraction of inspired oxygen, PEEP: Positive end-expiratory pressure, PSV: pressure support ventilation, ICU: intensive care unit, CV: current volume, NIV: noninvasive ventilation, *vs*.: versus; usual care^a^: respiratory physiotherapy, coughing exercises, IS, mobilization and nebulization with bronchodilator (2.5 mg of salbutamol every 6 hours), with saline solution (5 mL every 6 hours); CP^b^: diaphragmatic breathing exercises associated with active and/or active-assisted movement of the LLs and ULs, clearing maneuvers, coughing and re-expansion techniques; standard treatment^c^: oxygen, CP, intermittent nasal CPAP and pharmacological treatment.

### Risk of Bias

Of all the studies included in the systematic review, 30% described the random sequence generation^[8,15,17]^, 10% described allocation concealment^[15]^; none of the studies described blinding of the therapist and the patient, or presented this information; 40% of the studies described blindness of the outcome assessors, but for only one outcome in each study^[3,15,17,19]^. All studies described the losses and exclusions^[3,8,10,11,15-20]^, and 60% described the intention-to-treat analysis^[8,10,11,16,18,19]^ (Table 3).

**Table 3:** Risk of bias assessment.

Study, year	Random Sequence Generation	Allocation Concealment	Blinding Therapist	Blinding Patient	Blinding of the Outcome Assessment	Description of Losses and Exclusions	Intention-to-treat Analysis
Al Jaaly et al.^[15]^, 2013	Yes	Yes	No	Not informed	Yes*	Yes	No
Franco et al.^[16]^, 2011	Not informed	Not informed	Not informed	Not informed	Not informed	Yes	Yes
Jousela et al.^[11]^, 1994	Not informed	Not informed	Not informed	Not informed	Not informed	Yes	Yes
Lopes et al.^[8]^, 2008	Yes	Not informed	Not informed	Not informed	Not informed	Yes	Yes
Matte et al.^[3]^, 2000	Not informed	Not informed	Not informed	Not informed	Yes*	Yes	No
Mazullo et al.^[20]^, 2010	Not informed	Not informed	Not informed	Not informed	Not informed	Yes	No
Oikkonen et al.^[19]^, 1991	Not informed	Not informed	Not informed	Not informed	Yes**	Yes	Yes
Pinilla et al.^[17]^, 1990	Yes	Not informed	Not informed	Not informed	Yes**	Yes	No
Thomas et al.^[18]^, 1992	Not informed	Not informed	Not informed	Not informed	Not informed	Yes	Yes
Zarbock et al.^[10]^, 2009	Not informed	Not informed	Not informed	Not informed	Not informed	Yes	Yes

Yes*=for the Atelectasis outcome; Yes**= for the chest X-ray

### Intervention Effect

#### Mortality rate

Only one study (n=126) assessed the mortality rate. The authors compared the use of NIV associated with usual care (chest physiotherapy, bronchodilator and saline nebulization, cough exercises, mobilization and IS) *vs.* usual care. The mortality rate was the same in both groups (1.6%)^[15]^.

### Pulmonary Complications

#### Atelectasis

**Fig. 2:**
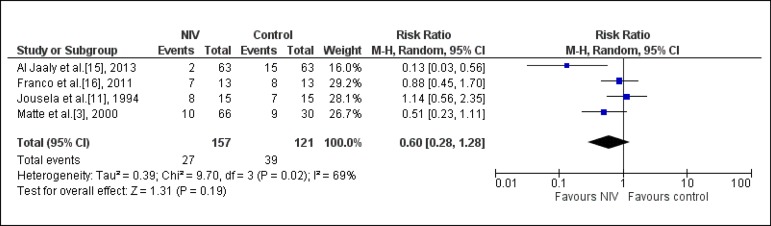
Analysis of the atelectasis regarding the studies that compared the prophylactic NIV to the control group. NIV=noninvasive ventilation

Six studies assessed the incidence of atelectasis^[3,11,15-17,19]^. Among them, four^[3,11,15,16]^ were included in the meta-analysis (n=407). One study compared NIV associated with usual care *vs.* usual care^[15]^; one compared NIV associated with conventional physiotherapy (CP) *vs.* CP^[16]^; another compared NIV *vs.* oxygen therapy, exclusively^[11]^; another study, with two intervention groups, compared NIV/CPAP mode associated with CP *vs.* CP; and NIV/BiLevel mode associated with CP *vs.* CP^[3]^. The use of NIV in the postoperative period of cardiac surgery did not significantly reduce the risk of atelectasis (RR: 0.60, CI95% 0.28, 1.28, I-square: 69%) (Figure 2).

This high statistical heterogeneity can be explained by a study by Al Jaaly et al.^[15]^, which presented a more favorable result to the use of NIV. One of the factors that may justify this has to do with the time of application of the therapy. The study applied the NIV for a longer period of time, and it was withdrawn when the patient had to eat, drink, or before if the patient could not tolerate the ventilation support, with average application time of 16 hours. In the other studies, the time was shorter (8 hours after extubation^[11]^, 1h every 3h, totaling 8h^[3]^, and 2 times a day for 30 minutes, totaling 1h^[16]^).

Other factors that could influence the effects of the NIV on the results are the ventilation parameters and type of intervention performed in the control group.

The other two studies^[17,19]^, which were not included in the meta-analysis due to lack of data, showed that the incidence of atelectasis decreased at the end of the intervention with NIV, but it did not differ from the control groups.

#### Pneumonia

**Fig. 3:**
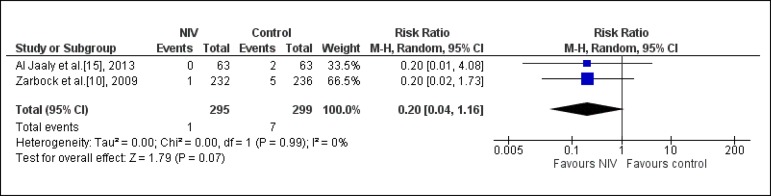
Analysis of the pneumonia regarding the studies that compared the prophylactic NIV to the control group. NIV=noninvasive ventilation

Two studies^[10,15]^ assessed this outcome (n=594). One study compared NIV associated to usual care *vs.* usual care^[15]^; and the other, NIV *vs.* standard treatment (oxygen therapy, CP, nasal intermittent CPAP for 10 min every 4h at 10 cmH_2_O and pharmacological treatment)^[10]^. From the analysis, we observed that the use of NIV did not significantly reduce the probability of pneumonia (RR: 0.20; CI95% 0.04; 1.16; I-square: 0%) (Figure 3).

#### Acute Respiratory Failure

Only one study assessed this outcome. Mazzulo Filho et al.^[20]^ carried out a study with 32 patients during the immediate postoperative period of cardiac surgery. The patients were randomly divided into two groups: control (n=18) and intervention (n=14), which received NIV/PSV mode during 2 hours, after extubation. As to the pulmonary complication, none of the patients from the intervention group presented ARF; on the other hand, nine patients from the control group did.

#### Hypoxemia

Three studies^[10,17,18]^ assessed this outcome. It was impossible to perform the meta-analysis of this outcome, because the studies did not present sufficient data for the analysis.

Zarbock et al.^[10]^ performed a study with 468 patients that underwent elective heart surgery, and compared the NIV/CPAP mode *vs.* standard treatment (oxygen therapy, CP, nasal intermittent CPAP for 10 min every 4h at 10 cmH_2_O and pharmacological treatment). The study showed that the incidence of hypoxemia (PaO_2_/FiO_2_< 100) was lower in the intervention group, when compared with the control group (1 of 232; 5 of 236 patients, respectively).

Pinilla et al.^[17]^ carried out a study with 58 patients, and compared NIV/CPAP mode associated to CP *vs.* CP and oxygen therapy. They found that the PaO_2_/FiO_2_ ratio was significantly better in the intervention group (*P*<0.05), half an hour until 24h after extubation, when compared with the control group; after that, it decreased in both groups (325±62, 320±37, in the intervention and control groups, respectively, without difference between groups).

Thomas et al.^[18]^ compared two groups with 14 patients after CABG, applying NIV/CPAP mode associated to CP *vs.* CP and oxygen therapy. The fraction of the pulmonary shunt was of 16.3% before, 12.6% during and 15.7% after the CPAP; in the control group, the shunt was reduced from 17.3% to 16.8%. This reduction was significantly higher in the CPAP group when compared with the control group (*P*=0.016).

### PaO_2_

Four studies^[3,8,11,19]^ assessed this outcome. It was not possible to perform the meta-analysis of this outcome, because the studies did not present sufficient data for the analysis.

Matte et al.^[3]^ performed a study with 96 patients, randomly divided into three groups. The study assessed two intervention groups: one group compared NIV/CPAP mode associated to CP (coughing, aerosol therapy, exercises, mobilization) *vs.* CP, and the other compared NIV/BiLevel mode associated to CP *vs.* CP. In the three groups, the PaO_2_ (mmHg) significantly decreased in the 1^st^ day of postoperative care (preoperative: control group 78±10, CPAP 76±12, BiLevel 81±10; 1^st^ day before treatment: control group 65±12, CPAP 63±9, BiLevel 66 ±11; *P*<0.001). For the patients of the control group, this decrease was still present on the 2^nd^ day; however, the patients in the intervention group presented a slightly improved PaO_2_ (*P*<0.01).

Lopes et al.^[8]^ developed a study with 100 patients that underwent CABG or heart valve surgery, randomly divided into two groups. The study applied NIV/BiLevel mode for 30 minutes after extubation *vs.* oxygen therapy. The NIV improved the PaO_2_, with significant difference (*P*=0.0009) between groups; the same happened with time, comparing the moment before extubation with 30, 120 and 360 minutes after the procedure (*P*=0.00008)^[8]^.

Conversely, two studies^[11,19]^ found different results. In a study performed with 30 patients that underwent CABG, who were randomly divided into two groups, NIV/CPAP mode *vs.* oxygen therapy, the PaO_2_ decreased significantly in the control group after extubation (from 19.2±5.3 kPa to 12.4±2.7 kPa), but it decreased less in the CPAP group (from 16.4±3.3 kPa to 14.0±2.1kPa). In the 2^nd^ PO, the PaO_2_ was equally low in both groups (control: 8.4±1.5 kPa, CPAP: 8.9±1.9 kPa)^[11]^.

Oikkonen et al.^[19]^ performed a study with 52 patients who were randomly divided into two groups: IPPB associated to CP *vs.* CP and IS. On the first three days of PO care, the values of PaO_2_ (kPa) were similar in both groups (1^st^ PO: control 14±1, IPPB 15±1; 2^nd^ PO: control 12±1, IPPB 11 ± 1; 3^rd^ PO: control 10±1, IPPB 11±1), without statistically significant differences. Based on this, both resources are equally efficient.

### Reintubation Rate

**Fig. 4:**
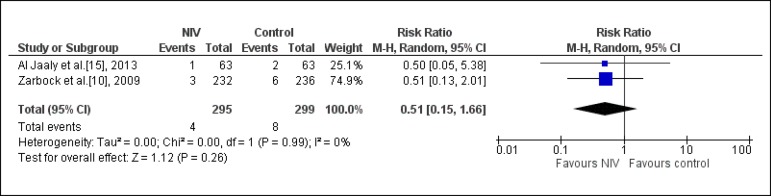
Analysis of the reintubation rate regarding the studies that compared the prophylactic NIV to the control group. NIV=noninvasive ventilation

Two studies assessed this outcome (n= 594)^[10,15]^. It was observed that using NIV does not significantly reduce the probability of reintubation (RR: 0.51; CI95%: 0.15; 1.66; I-square: 0%) (Figure 4).

### Time spent in the ICU

**Fig. 5:**
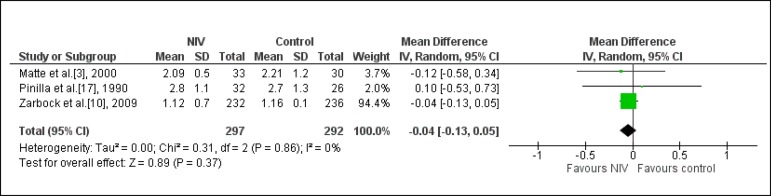
Analysis of the time spent in the intensive care unit regarding the studies that compared the prophylactic NIV to the control group. NIV=noninvasive ventilation.

Three studies assessed this outcome (n=641)^[3,10,17]^. One study assessed two intervention groups: one compared NIV/CPAP mode associated to CP *vs.* CP, and the other compared NIV/BiLevel mode associated to CP *vs.* CP^[3]^. Another study compared NIV associated to CP *vs.* oxygen therapy associated to CP^[17]^; and another study assessed NIV *vs.* standard treatment^[10]^. It was observed that using NIV does not significantly reduce the time spent in the ICU (-0.04 days; CI95%: - 0.13; 0.05; I-square: 0%) (Figure 5).

### Length of Hospital Stay

Only two studies assessed length of hospital stay (n=594)^[10,15]^, however, it was not possible to perform the meta-analysis due to lack of data. According to Al Jaaly et al.^[15]^, the length of hospital stay until release from hospital care, in days, was shorter in the intervention group, when compared with the control group (5±1.5; 6±1.5, respectively, *P*=0.019). Considering only the doctors' opinion, the patients could be released. However, because of non-medical reasons (such as social factors), the patients could not be released and the total length of hospital stay until the release was similar, without significant difference between groups (*P*=0.552).

A study by Zarbock et al.^[10]^ showed that the total length of hospital stay was similar between groups, without significant difference (intervention group 13±0.5 days, control group 14±0.6 days; *P*>0.05).

### Mechanical Ventilation Time

Three studies assessed this outcome (n=694)^[8,10,15]^; however, it was not possible to perform the meta-analysis due to lack of data.

In the study by Lopes et al.^[8]^, comparing NIV *vs.* oxygen therapy, the average time of mechanical ventilation was 3.77±0.94h, with no significant difference between the control and intervention groups (*P*=0.526). Zarbock et al.^[10]^ assessed NIV *vs.* standard treatment after admission to the ICU. The patients were divided into two groups: the late extubated group and the extubated group; after that, each group was subdivided into intervention and control groups. Patients in the late extubated group (intervention and control) were ventilated for the same length of time (6.2±0.5 h, 6.±0.7 h, respectively, *P*>0.05); data from the extubated group were not described. Finally, the third study assessed NIV associated with usual care *vs.* usual care, and the average time was 6 hours for the intervention and control groups^[15]^.

## DISCUSSION

### Evidence Summary

The objective of this study was to search for the best available scientific evidence regarding the prophylactic use of NIV in patients during immediate postoperative care for cardiac surgery. Regarding the mortality rate, only one study assessed this outcome, and the result was similar between the intervention and control groups. We observed that prophylactic NIV, when compared to conventional physiotherapy or oxygen therapy, did not significantly reduce the probability of pulmonary complications such as atelectasis, pneumonia, reintubation rate and time spent in the ICU. As to the outcome of ARF, the incidence was higher in the control group. Regarding the outcome of hypoxemia, in most studies, the use of NIV improved oxygenation. Regarding PaO_2_, only half of the studies that assessed this outcome found that NIV improved it. As to the mechanical ventilation time and length of hospital stay, the results were similar between the groups.

For the outcome mortality rate, study showed low incidence between the intervention and control groups. This can be explained by the fact that the patients included in the studies had preoperative comorbidities, and similar surgical characteristics, among others, without the need for emergency surgery^[15]^. However, more studies should be carried out to expand this information.

Another outcome assessed by our study was pulmonary complications. These are frequent in the PO of cardiac surgery, and the etiology of the dysfunction results from a multifactorial process, which involves surgical and preoperative factors, well reported in the literature^[4-7]^. Thus, early therapy is necessary to avoid further degradation^[3]^.

From the review, it was observed that using NIV during the postoperative care of heart surgery did not significantly reduce the risk of atelectasis, but it improved oxygenation. This complication is often caused by a compromised ventilation lung perfusion ratio due to atelectasis. Even after cardiac surgery without complications, atelectatic areas decrease functional residual capacity and increase pulmonary shunt. These unventilated areas can account for up to 20% of the total lung volume, thus causing hypoxemia in the PO^[10]^. In this sense, NIV avoids alveolar collapse and enables better alveolar recruitment, reducing the formation of atelectasis and increasing functional residual capacity^[10,16]^.

Even though NIV presents positive effects in other populations, such as patients with ARF in the postoperative period of abdominal surgery^[21]^, in our study we have not yet found a beneficial effect of this intervention in relation to atelectasis. One thing that may justify these unfavorable outcomes are the NIV pressure parameters, with CPAP values between 5 and 7.5 cmH_2_O^[3,11]^. These pressures low in the airways have transitory effects on gas exchange^[10,17]^. It is believed that, for a prolonged effect, high pressure values are required to keep airways open^[10]^. A previous study performed with patients after thoracic surgery demonstrated that pressures of at least 9 to 10 cmH_2_O should be used to maintain positive tracheal pressure throughout the respiratory cycle^[22]^.

Another pulmonary complication is pneumonia. From the analysis, it was observed that using NIV did not significantly reduce the probability of pneumonia^[10,15]^. We observed that there is a favorable tendency to the use of NIV; however, more RCTs, with larger sample sizes, are necessary to corroborate this information.

Regarding the outcomes of reintubation rate and time spent in the ICU, it was observed that using NIV does not significantly reduce the probability of reintubation and the time spent in the ICU. This may have occurred because of the small number of included studies (two and three, respectively). It is possible that with more RCTs, with larger sample sizes, this result could change; therefore, there is no evidence on the effectiveness of NIV on the reintubation rate and time spent in the ICU.

Another assessed outcome was the length of hospital stay, but only two studies assessed it^[10,15]^. The time was similar between groups in both studies, with no significant difference between groups. This similarity can be justified by the low incidence of pulmonary complications in both studies, which could prolong this time if an increase occurred.

### The Strengths and Limitations of the Study

Regarding the strong methodological points of this study, it is important to point out the systematic and sensitive bibliographic search, with explicit and reproducible eligibility criteria, without restriction of language and date, independently performed by two assessors; as well as the selection of studies, data extraction and analysis of methodological quality of included articles, also performed independently by two assessors. In addition to that, a meta-analysis was performed with the results of the studies, provided they allowed such analysis, since the meta-analysis can give more reliable estimates as to the efficacy of the treatment.

As to the limitations, the low methodological quality of the included studies stands out, since the indispensable items for assessing the risk of bias were presented incompletely or not informed. In addition, the included studies were quite different regarding the physiotherapy techniques, resources and exercises used, time of intervention, time of application of NIV and frequency of examinations. All this may compromise the results found in the meta-analyses.

In addition to the methodological differences, we highlight the small number of studies found in the literature, and the sample size, with small number of patients, which suggest the need for new RCTs with more patients and more methodological rigor.

## CONCLUSION

Our study showed that no difference between the use of prophylactic NIV and conventional physiotherapy or oxygen therapy could be found in patients during the postoperative period of cardiac surgery, in relation to mortality rate and pulmonary complications such as atelectasis, pneumonia, reintubation rate, time spent in the ICU, length of hospital stay and mechanical ventilation time, with an improvement in oxygenation. Therefore, due to the low methodological rigor of the included articles and small sample size, new RCTs should be carried out to corroborate this information.

**Table:** 

Authors' roles & responsibilities	
SMP	Conception and design of the work; acquisition, analysis, interpretation of data for the work; drafting the work and revising it critically for important intellectual content; final approval of the version to be published
AGFM	Conception and design of the work; acquisition, analysis, interpretation of data for the work; drafting the work and revising it critically for important intellectual content; final approval of the version to be published
GS	Conception and design of the work; acquisition, analysis, interpretation of data for the work; drafting the work and revising it critically for important intellectual content; final approval of the version to be published
